# The Association of Meat Intake With All-Cause Mortality and Acute Myocardial Infarction Is Age-Dependent in Patients With Stable Angina Pectoris

**DOI:** 10.3389/fnut.2021.642612

**Published:** 2021-03-04

**Authors:** Åslaug O. Matre, Anthea Van Parys, Thomas Olsen, Teresa R. Haugsgjerd, Carl M. Baravelli, Ottar Nygård, Jutta Dierkes, Vegard Lysne

**Affiliations:** ^1^Department of Clinical Science, Centre for Nutrition, University of Bergen, Bergen, Norway; ^2^Mohn Nutrition Research Laboratory, Centre for Nutrition, University of Bergen, Bergen, Norway; ^3^Department of Nutrition, Institute of Basic Medical Sciences, University of Oslo, Oslo, Norway; ^4^Department of Global Public Health and Primary Care, University of Bergen, Bergen, Norway; ^5^Department of Laboratory Medicine and Pathology, Haukeland University Hospital, Bergen, Norway; ^6^Department of Heart Disease, Haukeland University Hospital, Bergen, Norway; ^7^Department of Clinical Medicine, University of Bergen, Bergen, Norway

**Keywords:** meat, acute myocardial infarction, cancer, mortality, effect modification, gastrointestinal cancer, stable angina pectoris, cardiovascular disease

## Abstract

**Background:** Red and processed meat intake have been associated with increased risk of morbidity and mortality, and a restricted intake is encouraged in patients with cardiovascular disease. However, evidence on the association between total meat intake and clinical outcomes in this patient group is lacking.

**Objectives:** To investigate the association between total meat intake and risk of all-cause mortality, acute myocardial infarction, cancer, and gastrointestinal cancer in patients with stable angina pectoris. We also investigated whether age modified these associations.

**Materials and Methods:** This prospective cohort study consisted of 1,929 patients (80% male, mean age 62 years) with stable angina pectoris from the Western Norway B-Vitamin Intervention Trial. Dietary assessment was performed by the administration of a semi-quantitative food frequency questionnaire. Cox proportional hazards models were used to investigate the association between a relative increase in total meat intake and the outcomes of interest.

**Results:** The association per 50 g/1,000 kcal higher intake of total meat with morbidity and mortality were generally inconclusive but indicated an increased risk of acute myocardial infarction [HR: 1.26 (95% CI: 0.98, 1.61)] and gastrointestinal cancer [1.23 (0.70, 2.16)]. However, we observed a clear effect modification by age, where total meat intake was associated with an increased risk of mortality and acute myocardial infarction among younger individuals, but an attenuation, and even reversal of the risk association with increasing age.

**Conclusion:** Our findings support the current dietary guidelines emphasizing a restricted meat intake in cardiovascular disease patients but highlights the need for further research on the association between meat intake and health outcomes in elderly populations. Future studies should investigate different types of meat separately in other CVD-cohorts, in different age-groups, as well as in the general population.

## Introduction

Meat consumption has increased considerably worldwide in the last decades (1). Meat is the edible portion of animals and includes muscles, fats, tendons, ligaments, and offal. Meat can be classified as red or white; red meat usually includes meat from cattle, pigs, and sheep, whereas white meat includes meat from poultry (2). As a food group, meat is an important source of several essential nutrients, including protein, thiamin, niacin, vitamin B-6 and B-12, zinc, selenium, and heme iron. Red meat is also a major source of saturated fatty acids, and processed meat products are major contributors to salt intake (3). High consumption of red and processed meat has been associated with increased risk of several adverse health outcomes, including overweight and obesity (4), type 2 diabetes mellitus (5), cardiovascular disease (CVD) (6), cancer (7), and all-cause mortality (8) in initially healthy populations. Particularly, dietary intakes of red and processed meat have gained attention for their association with increased colorectal cancer risk (9). In contrast, less evidence has been accrued with respect to populations with pre-existing disease. Nevertheless, the World Cancer Research Fund recommends restricting the consumption of red and processed meat (10), which has become a cornerstone of dietary guidelines in the Western world (11).

Current dietary guidelines to reduce the risk of CVD also emphasize reduced intake of dietary sources of trans- and saturated fatty acids, including red and processed meat (12). However, data on the association between meat intake and clinical outcomes among CVD patients are lacking. In this study, we aimed to investigate the association between total meat intake and risk of all-cause mortality, and incident acute myocardial infarction (AMI), cancer, and gastrointestinal (GI)-cancer in patients with established CVD. Furthermore, because meat intake can contribute to energy and nutrient sufficiency in an aging population prone to malnutrition (13) we also investigated the potential effect modification by age.

## Subjects and Methods

### Study Cohort

**Figure 1:**
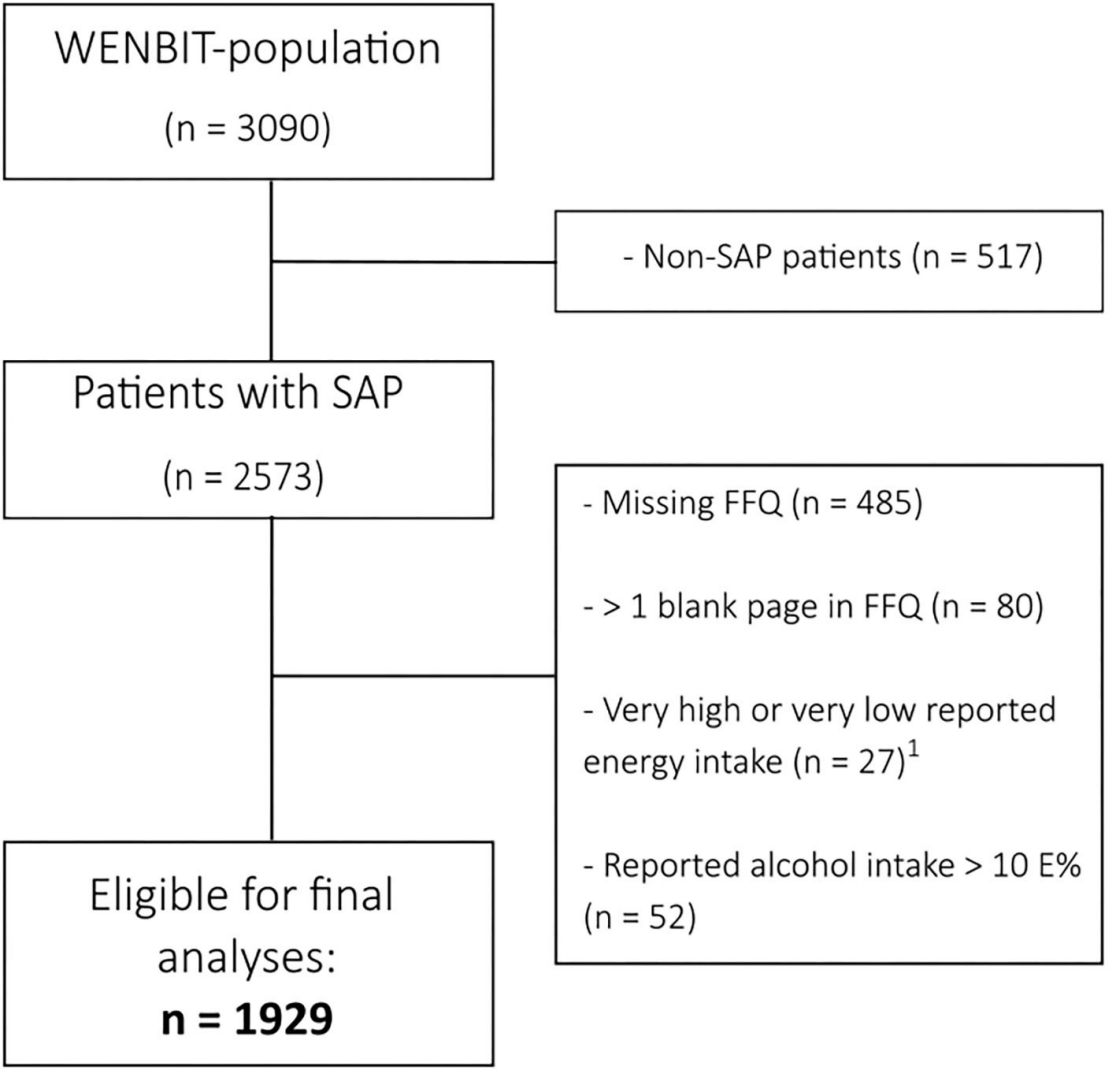
Flowchart illustrating the selection of patients from the WENBIT-cohort. ^1^Self-reported energy intake <717 kcal or >3,585 kcal (<3,000 or >15,000 kJ) for females, and <789 or >4,183 kcal (<3,300 or >17,500 kJ) for males. FFQ, Food frequency questionnaire; SAP, Stable angina pectoris; WENBIT, Western Norway B-vitamin Intervention Trial.

The population under study consisted of 1,929 patients (80% male, mean age 62 years) who were participating in the Western Norway B-vitamin Intervention Trial (WENBIT, NCT00354081). A total of 3,090 patients were included in WENBIT, which was a randomized, double-blind, placebo-controlled trial conducted between 1999 and 2004 investigating the effect of treatment of homocysteine-lowering B-vitamins on mortality and cardiovascular outcomes. Inclusion criteria for participation in the trial were males and females >18 years with suspected stable angina pectoris who were undergoing coronary angiography at Haukeland University Hospital (Bergen, Norway) or Stavanger University Hospital (Stavanger, Norway). Exclusion criteria were unavailability for follow-up, participation in other trials, known alcohol abuse, severe mental illness, or cancer. The trial has been described in detail elsewhere (14, 15). For the current analyses, we included only patients with confirmed stable angina pectoris (*n* = 2,573). We excluded patients with missing (*n* = 485) or incomplete (>1 blank page in the FFQ, *n* = 80) dietary data, and implausible reporters using simple criteria (16) (self-reported energy intake <717 kcal or >3,585 kcal (<3,000 or >15,000 kJ) for females, and <789 or >4,183 kcal (<3,300 or >17,500 kJ) for males, *n* = 27). We also excluded patients with a very high reported alcohol intake (>10 E%, *n* = 52), leaving a total of 1,929 patients eligible for analyses. A flowchart illustrating the inclusion process is provided in Figure 1.

### Data Sources

Data on lifestyle factors and medical history were obtained from self-reported questionnaires and verified by hospital records. Patients were defined as smokers if they reported being current smokers, had quit within the last 4 weeks, or had plasma cotinine levels >85 nmol/L. Diabetes mellitus was defined according to preexisting diagnosis, plasma HbA1c >6.5%, fasting blood glucose >7 mmol/L or non-fasting blood glucose >11.1 mmol/L. Blood samples and routine laboratory analyses were performed at the recruiting hospitals using standard methods.

Dietary data were obtained from a self-administered semi-quantitative food frequency questionnaire (FFQ), which was given at the first visit and returned at the 1-month follow-up visit, or by mail. The FFQ was designed to capture the habitual Norwegian diet and consisted of 169 food items grouped according to Norwegian meal patterns. Patients were asked to answer the questionnaire based on their food habits the year before the study. The data were converted from portion sizes and household measures into grams per day. Nutrient intake was calculated by using “Kostberegningssystemet” version 3.2, developed by the Department of Nutrition, University of Oslo, Norway.

### Ethical Considerations

The study was carried out according to the Declaration of Helsinki and was approved by The Regional Committee for Health Research Ethics and The Norwegian Data Inspectorate. All patients provided written informed consent.

### Exposure

Due to the FFQ used for dietary assessment, it was not possible to distinguish between different types of meat. Accordingly, the exposure in this study was total meat intake. For cold cuts, the frequency of consumption was given as times per week, and portion sizes were given as per slice of bread. For meat products used in dishes, the frequency was given as time per month, and portion sizes as slices, pieces, or by volume.

### Clinical Endpoints

All-cause mortality, AMI, cancer, and GI-cancer were chosen as primary outcomes of interest. AMI included both fatal- and non-fatal AMI (International Classification of Diseases (ICD)-10 codes: I21, I22, I46.1, R96, R98). Cancer covered malignant neoplasms of all sites (ICD-10 codes: C00–C97), whereas GI-cancer included malignant neoplasms in the GI-tract, and accessory organs of digestion, including liver, gallbladder, and pancreas (ICD-10 codes: C15–C26). Information on mortality was obtained from the Cause of Death Registry at the Norwegian Institute of Public Health (17), information on AMI was obtained from the Cardiovascular disease in Norway-project (18), while information on cancer was obtained from the Cancer Registry of Norway (19).

### Statistical Analyses

**Figure 2:**
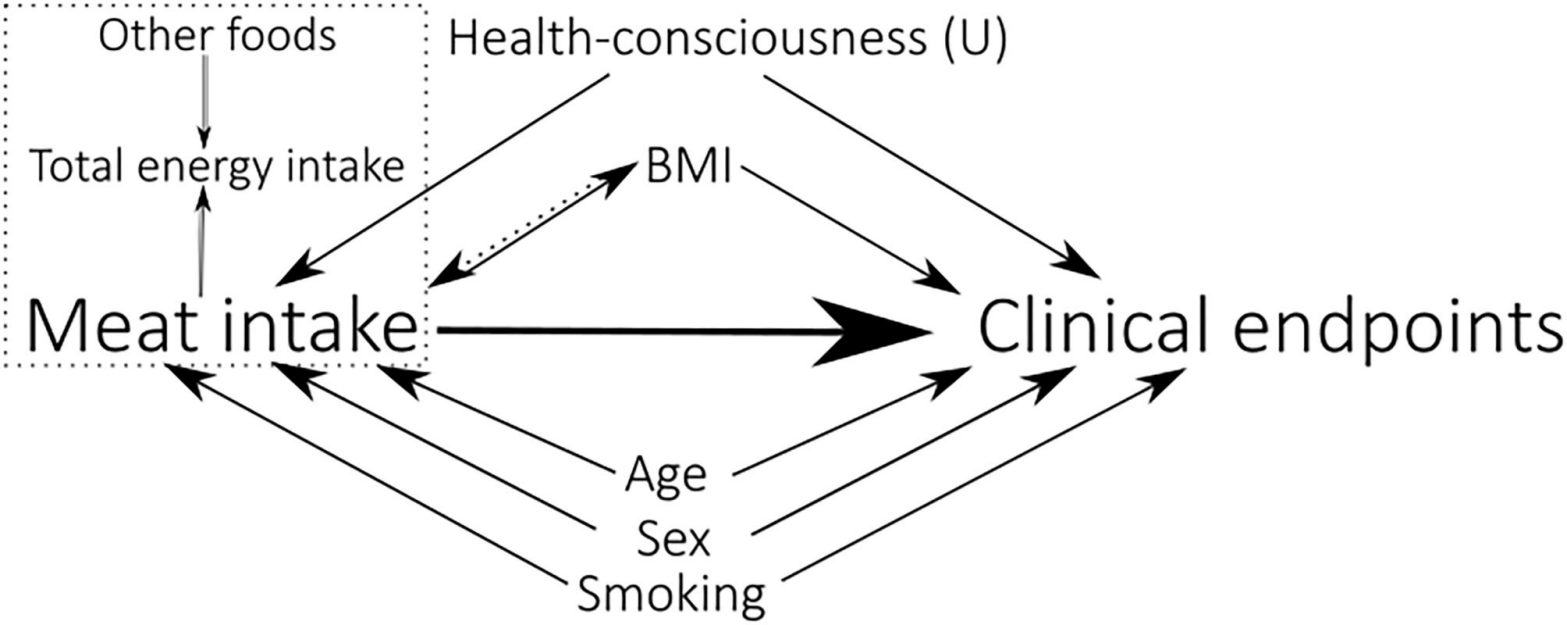
A directed acyclic graph illustrating the model building process, representing the causal assumptions that were made. The variables pointing toward both Meat intake and Clinical endpoints (all-cause mortality, AMI, cancer, and GI-cancer), are considered confounders for these associations. The (U) indicates that the variable is unmeasured. BMI is modeled both as a mediator (Model 1) and a confounder (Model 2). A dashed rectangle encompasses variables occurring at the same time.

All statistical analyses were performed using R, version 4.0.2 (The R Foundation for Statistical Computing, Vienna, Austria), and the packages within the Tidyverse (*dplyr, tidyr, broom, purrr, stringr, rlang, and ggplot2*) (20), *skimr, survival*, and *ggridges*. The analytic code is available in the Supplementary Material. To adjust for self-reported energy intake, the nutrient density method was used (21), expressing foods as g/1,000 kcal and macronutrients as energy % (E%). Cox proportional hazard regression models, with days since baseline as time scale, were used to investigate the association between meat intake and the outcomes of interest, and the HRs are reported per 50 g/1,000 kcal increment in daily meat intake. The proportional hazard assumption was tested using the *cox.zph()* function in the *survival* package, and by visually inspecting Schoenfeld residuals. Confounding variables were identified *a priori*, based on subject matter knowledge at the time of writing and previous literature, using a directed acyclic graph approach (Figure 2). To estimate the relative effect of increasing meat intake, self-reported total energy intake was included as a covariate in all models. The HR is then interpreted as the change in the estimated risk for each 50 g/1,000 kcal increment in meat intake, with a concomitant isoenergetic decrease in intake of other foods (22). The main model (Model 1) included age, sex, and smoking as covariates. Model 2 was additionally adjusted for BMI. Model 3 and 4 were adjusted for the same covariates as Model 1 and 2, respectively, except age and BMI being included as penalized splines (using the *pspline* function in the *survival* package). The same models were applied to all outcomes. The analyses were also conducted for males and females separately and presented in Supplementary Table 3.

**Figure 3:**
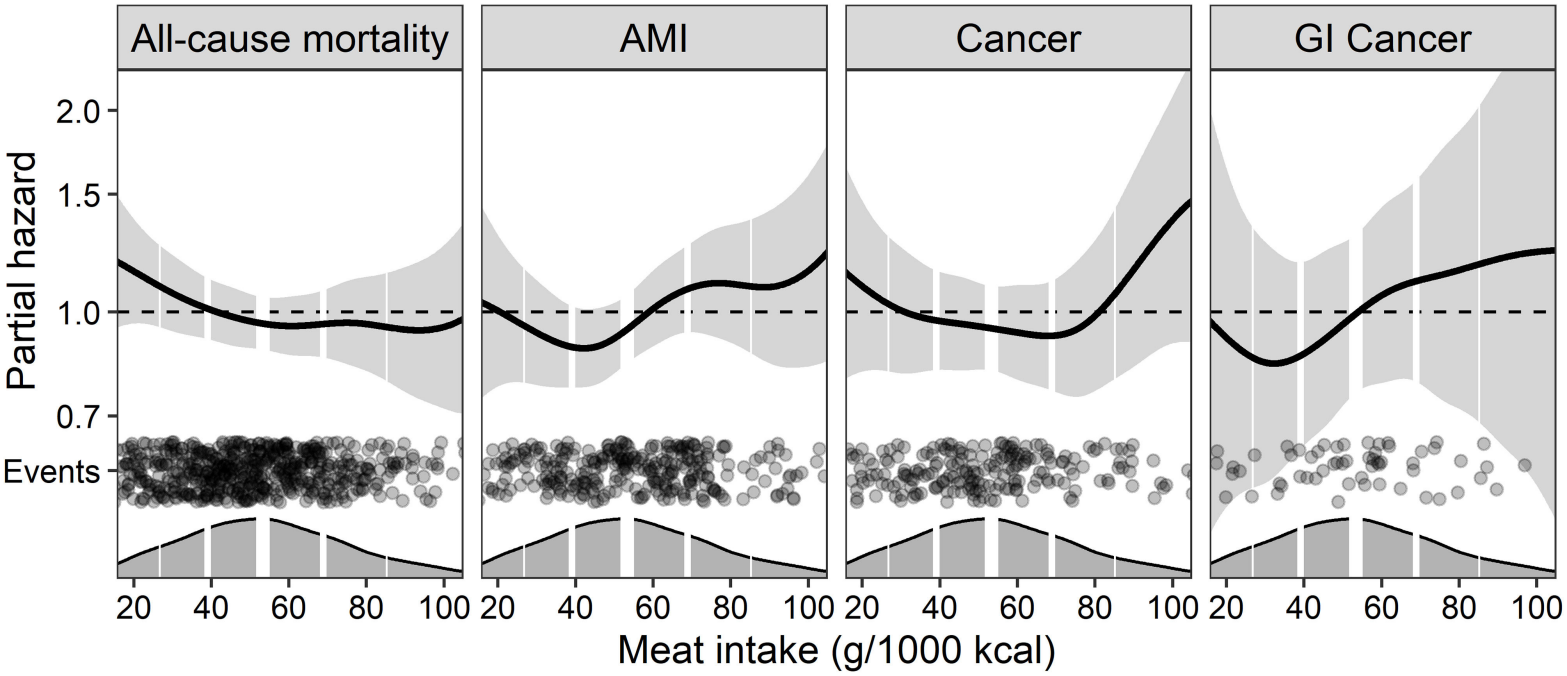
The continuous association between energy-adjusted total meat intake and the risk of clinical outcomes using generalized additive models. The models were adjusted for Model 1 covariates (self-reported energy intake, age, sex, and smoking). Light gray areas around the central line represent 95% CIs of the hazard estimates. Dark gray areas at the x-axis are density plots of meat intake (g/1,000 kcal), and the vertical white lines indicate the 10th, 25th, 50th (bold line), 75th, and 90th percentiles. The black dots indicate events at the different intake levels of meat. The plots are cropped at the 2.5th and 97.5th percentiles of reported meat intake. AMI, Acute myocardial infarction; GI, Gastrointestinal.

**Figure 4:**
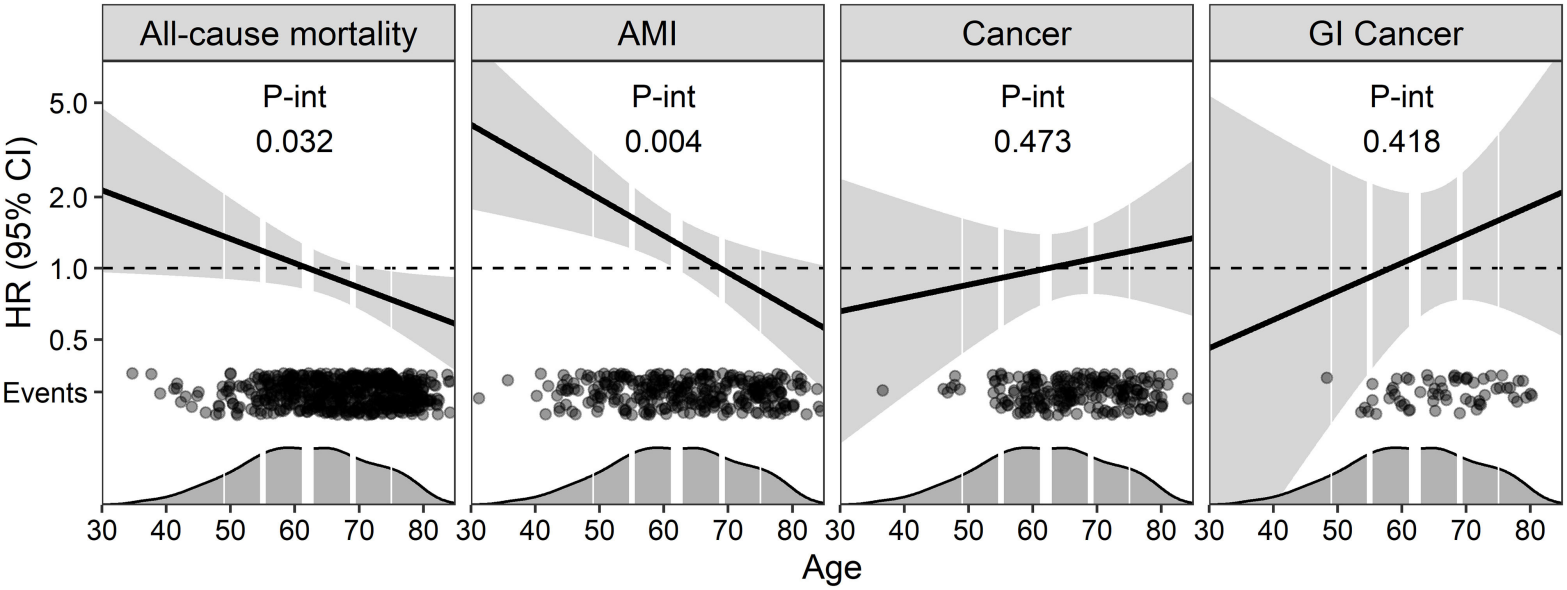
The association between energy-adjusted total meat intake and the risk of clinical outcomes across age. The curves are fitted by including age as an interaction term to Model 1 (adjusted for self-reported energy intake, age, sex, and smoking). The HRs are given per 50 g/1,000 kcal higher intake of meat, and light gray areas around the central line represent 95% CIs of the hazard ratios. Dark gray areas at the x-axis are density plots of age, and the vertical white lines indicate the 10th, 25th, 50th (bold line), 75th, and 90th percentiles. The black dots indicate events across age. AMI, Acute myocardial infarction; GI, Gastrointestinal.

To explore potential non-linear relationships, meat intake was modeled as a penalized spline in a model adjusted for Model 1 (Figure 3) and Model 2 (Supplementary Figure 1) covariates, and the partial hazard (95% CI) of increasing meat intake was visualized. We further explored whether age modified the association for meat intake with the endpoints, by including an interaction term to Model 1 (Figure 4) and Model 2 (Supplementary Figure 2). The conditional HRs (95% CI) per 50 g/1,000 kcal higher intake of meat was then plotted across age to visualize the potential effect modification. Models with and without interaction terms were compared by likelihood ratio tests and presented in Supplementary Table 4.

In line with the most recent statement from the American Statistical Association on *p*-values (23), we decided not to dichotomize the results based on *p*-value cutoffs. We rather describe our data emphasizing effect sizes, the variation, and uncertainty of the data as expressed by CIs (24). The importance and practical implications of the observations were evaluated using subject matter knowledge and prior evidence.

## Results

### Patient Characteristics

Key characteristics of the 1,929 patients and the associations with meat intake are presented in Table 1, with a more complete description of the study population provided in Supplementary Table 1. In the final cohort of 1,929 patients, 1,539 patients (79.8%) were male, and the mean ± SD age was 62 ± 10 years. A total of 836 patients (43.3%) had experienced an AMI previously. Patients with a relatively higher meat intake were more likely to be younger, male, smokers, diabetic, and to have a higher BMI and larger waist circumference.

**Table 1:** Key characteristics of the patients included in the study (*n* = 1,929).

**Variable**	**Total cohort^a^**	**Percentiles**					**Association with meat intake^b^**
		**10**	**25**	**50**	**75**	**90**	
Age, years	61.8 ± 9.7	49.0	55.0	62.0	69.0	75.0	r = −0.20 (−0.24, −0.16)
BMI, kg/m^2^	26.4 ± 3.7	22.0	24.0	26.0	28.0	31.0	r = 0.14 (0.10, 0.18)
Waist circumference, cm	96.4 ± 11.1	83.0	90.0	96.0	103	110	r = 0.12 (0.08, 0.17)
Male sex	1,539 (79.8%)	–	–	–	–	–	d = 3.6 (1.1, 6.1)
Hypertension	911 (47.2%)	–	–	–	–	–	d = −0.4 (−2.5, 1.6)
Diabetes^c^	590 (30.6%)	–	–	–	–	–	d = 3.5 (1.3, 5.7)
Smoker^d^	560 (29.0%)	–	–	–	–	–	d = 6.4 (4.2, 8.7)

a *Presented as mean ± SD or n (%)*.

b *Given as d = mean difference (95% CI), or r = Pearson's correlation coefficient (95% CI), calculated on log transformed data*.

c *Preexisting diagnose of diabetes, or with fasting blood glucose >7 mmol/L, non-fasting blood-glucose >11.1 mmol/L, or HbA1c >6.5%*.

d *Self-reported current smokers, or having quit within the last 4 weeks, or with plasma cotinine levels ≥85 nmol/L*.

### Dietary Intake

The daily dietary intake of the macronutrients (given in E%) and food groups (given in g/1,000 kcal), and their associations with meat intake are provided in Table 2. A more complete description of the dietary intake is provided in Supplementary Table 2. The mean daily reported meat intake was 54.9 ± 22.9 g/1,000 kcal (absolute intakes: 123 ± 62 g and 86 ± 46 g for males and females, respectively). Relatively higher meat intake was associated with slightly lower consumptions of dairy products, fruits and berries, and bread. As expected, given the nutrient content of meat, relatively higher meat intake was positively associated with intake of fats (both saturated-, monounsaturated-, and polyunsaturated fats), protein, and cholesterol, and inversely associated with carbohydrate intake.

**Table 2:** The daily dietary intake of the patients (*n* = 1,929).

**Intake**	**Mean ± SD**	**Null consumers (%)**	**Percentiles**					**Association with meat intake^a^**
			**10**	**25**	**50**	**75**	**90**	
**Macronutrients (E%)**								
Fat	32.0 ± 5.5	–	24.9	28.4	31.8	35.7	38.9	r = 0.29 (0.25, 0.33)
SFA	11.8 ± 2.6	–	8.56	10.0	11.6	13.3	15.0	r = 0.23 (0.19, 0.27)
MUFA	10.3 ± 2.0	–	7.86	8.97	10.3	11.6	12.8	r = 0.38 (0.35, 0.42)
PUFA	7.22 ± 2.0	–	5.01	5.76	6.90	8.40	9.82	r = 0.12 (0.07, 0.16)
Carbohydrate	49.1 ± 6.2	–	41.3	45.1	49.3	53.4	56.7	r = −0.36 (−0.40, −0.32)
Protein	16.7 ± 2.5	–	13.7	15.0	16.5	18.2	19.9	r = 0.24 (0.20, 0.29)
Alcohol	1.70 ± 2.1	493 (25.6%)	0.00	0.00	0.96	2.70	4.72	r = 0.12 (0.08, 0.17)
**Food groups, g/1,000 kcal**								
Meat	54.9 ± 22.9	1 (0.1%)	26.7	39.0	53.3	68.9	85.1	–
Egg	8.34 ± 6.12	42 (2.2%)	1.96	4.03	7.15	11.0	15.9	r = 0.07 (0.03, 0.12)
Dairy	158 ± 108	1 (0.1%)	31.1	73.8	143	221	302	r = −0.09 (−0.13, −0.05)
Milk	133 ±107	10 (0.5%)	11.3	41.1	118	194	271	r = −0.05 (−0.10, −0.01)
Cheese	13.3 ± 11.8	141 (7.3%)	1.02	5.07	10.3	18.2	28.9	r = 0.00 (−0.05, 0.04)
Bread	92.6 ± 31.9	10 (0.5%)	54.2	71.5	90.4	112	135	r = −0.09 (−0.13, −0.05)
Grains	15.8 ± 13.1	11 (0.6%)	3.25	6.73	12.8	21.3	31.0	r = 0.02 (−0.02, 0.07)
Fish	53.7 ± 28.5	5 (0.3%)	21.9	33.6	48.8	68.7	91.6	r = −0.03 (−0.08, 0.01)
Vegetables	106 ± 75.0	2 (0.1%)	37.4	57.5	88.7	134	189	r = 0.08 (0.04, 0.13)
Fruits and berries	126 ± 86.0	3 (0.2%)	40.9	67.9	108	163	230	r = −0.11 (−0.15, −0.06)
**Other**								
Energy, kcal	2,092 ± 631	–	1,320	1,653	2,033	2,478	2,953	r = 0.06 (0.02, 0.11)
Fiber, g/1,000 kcal	25.2 ± 8.6	–	15.1	19.1	24.4	30.0	36.3	r = −0.11 (−0.15, −0.06)
Cholesterol, mg/1,000 kcal	142 ± 39	–	98.2	116	138	162	189	r = 0.28 (0.24, 0.32)

a *Given as r = Pearson's correlation coefficient (95% CI), calculated on log-transformed data*.

*MUFA, Monounsaturated fatty acid; PUFA, Polyunsaturated fatty acid; SFA, Saturated fatty acid*.

### Meat Intake and Risk of Clinical Endpoints

#### All-Cause Mortality

A total of 572 (29.8%) patients died after a median follow-up of 14.1 (25th, 75th percentiles: 12.8, 15.5) years. In Model 1, we observed a 9% reduced risk of all-cause mortality [HR: 0.91 (95% CI: 0.75, 1.10)] per 50 g/1,000 kcal higher intake of meat, but the data was compatible with both 25% reduced risk and 10% increased risk. Further adjustments did not have any pronounced impact on the result (Table 3), but the association appeared stronger in females (Supplementary Table 3). The association between meat intake and all-cause mortality was age-dependent, with an increased risk observed at younger ages, but an attenuation and even reversal of the risk association observed with increasing age (Figure 4). Including age as an interaction term improved model fit (Supplementary Table 4).

**Table 3:** The association between energy-adjusted total meat intake and clinical endpoints—results from the Cox proportional hazard regression model^a^.

	**All-cause mortality**		**AMI**		**Cancer**		**GI-cancer**	
Number of events (%)	574 (29.8%)		309 (16.0%)		213 (11.0%)		61 (3.2%)	
Median follow-up, years (25th, 75th percentile)	14.1 (12.8, 15.5)		7.8 (6.4, 9.1)		8.0 (6.6, 9.3)		8.1 (6.7, 9.4)	
**Models^b^**	**HR (95% CI)**	***P*****-value**	**HR (95% CI)**	***P*****-value**	**HR (95% CI)**	***P*****-value**	**HR (95% CI)**	***P*****-value**
Model 1	0.91 (0.75, 1.10)	0.333	1.26 (0.98, 1.61)	0.077	1.04 (0.76, 1.42)	0.806	1.23 (0.70, 2.16)	0.478
Model 2	0.88 (0.72, 1.07)	0.189	1.21 (0.93, 1.56)	0.150	1.03 (0.76, 1.42)	0.832	1.16 (0.65, 2.06)	0.613
Model 3	0.89 (0.73, 1.08)	0.247	1.23 (0.95, 1.59)	0.109	1.05 (0.77, 1.43)	0.750	1.25 (0.71, 2.19)	0.444
Model 4	0.88 (0.72, 1.07)	0.202	1.20 (0.93, 1.56)	0.164	1.05 (0.77, 1.43)	0.772	1.17 (0.66, 2.08)	0.594

a *HRs (95% CI) are given per daily 50 g/1,000 kcal higher intake of meat*.

b *Model 1: Adjusted for age, sex, smoking, and total energy intake. Model 2: Adjusted for age, sex, smoking, BMI, and total energy intake. Model 3: Adjusted for age (spline), sex, smoking, and total energy intake. Model 4: Adjusted for age (spline), sex, smoking, BMI (spline), and total energy intake. AMI, Acute myocardial infarction; GI, Gastrointestinal*.

#### Acute Myocardial Infarction

During the follow-up of 7.8 (25th, 75th percentiles: 6.4, 9.1) years, 309 (16.0%) incident cases of AMI were reported. In Model 1, meat intake was associated with a 26% increased risk of AMI [1.26 (0.98, 1.61)], per 50 g/1,000 kcal higher intake of meat (Table 3). The relationship between meat intake and AMI appeared non-linear, with the increased risk mainly observed in the higher intake ranges of meat (Figure 3). The increased risk of AMI appeared to be present at younger ages, but an attenuation of the risk association with increasing age (Figure 4). Including age as an interaction term improved model fit (Supplementary Table 4).

#### Cancer

In total, 213 (11.0%) patients were diagnosed with cancer during the follow-up of 8.0 (25th, 75th percentiles: 6.6, 9.3) years. In Model 1, meat intake was associated with a 4% increased risk of cancer per 50 g/1,000 kcal higher intake of meat per day. However, the low precision rendered the data inconclusive [1.04 (0.76, 1.42)]. Further adjustments did not appreciably affect the results (Table 3). Continuous analyses indicated a non-linear association with cancer, with an increased risk of cancer at higher intake-levels of meat, but not at intakes in the low- to moderate range (Figure 3).

A total of 61 (3.2%) patients were diagnosed with GI-cancer during follow-up of 8.1 (25th, 75th percentiles: 6.7, 9.4) years. In Model 1, we observed a 23% increased risk of GI-cancer per 50 g/1,000 kcal higher intake of meat [1.23 (0.70, 2.16)], but the data was compatible both with 30% reduced risk and 116% increased risk of GI-cancer. Including BMI as a covariate in Model 2 and Model 4 slightly attenuated the association (Table 3). The association with GI-cancer appeared slightly J-shaped (Figure 3).

## Discussion

### Main Findings

In this study, the association of a higher meat intake with morbidity and mortality were generally inconclusive but indicated an increased risk of AMI and GI-cancer. However, we observed a clear effect modification by age, where total meat intake was associated with an increased risk of mortality and AMI at younger ages, but an attenuation and even reversal of the risk association observed with increasing age.

### Discussion of the Findings

Our findings on AMI and GI-cancer are supported by previously reported findings on red and processed meat in the existing literature based on initially healthy populations (9, 25), and provide support to the current dietary guidelines emphasizing a restricted meat consumption in the Western world, in particular at younger ages. However, the reduced risk we observed for all-cause mortality contradicts previously reported findings (8). This unexpected association may partly be explained by the observation of a strong inverse association between meat intake and age in this study population, with the oldest participants having the lowest meat intake. Furthermore, we observed that the association between meat intake and mortality and acute myocardial infarction was different across age, with increased risk in the younger end of the population, but reduced risk at higher ages. Older populations typically have a lower dietary intake and a higher prevalence of undernutrition compared to younger populations (26), which is associated with an increased risk of mortality (27). In addition to contributing to energy intake, meat can be an important source of essential micronutrients and protein of high biological value. Increased protein intake has previously been associated with improved health in older populations (28). Thus, we may speculate that a higher meat intake may be more beneficial, or less harmful, in elderly populations compared to younger populations. Key et al. (25) reported an association between red and processed meat, and increased risk of myocardial infarction in participants <65 years from the EPIC (European Prospective Investigation into Cancer and Nutrition) cohort. However, the results indicated a reduced risk among the participants ≥65 years, supporting the current findings on AMI. Furthermore, Levine et al. (29) reported an association between moderate to high protein intakes, and increased risk of all-cause mortality among participants aged 50–65 years in a United States population. When they controlled for the E% from animal protein, this association diminished, suggesting that the observed association was driven by high intakes of animal protein. However, among participants aged ≥66 years, they observed that higher protein intake was associated with reduced risk of all-cause mortality, without controlling for animal protein, which is similar to the current observations with meat intake. Conversely, Letois et al. (30) reported an increased risk of mortality associated with increasing meat intake in a French population aged ≥65 years. Further research on the association between meat intake and health outcomes in elderly populations is needed.

### Suggested Underlying Mechanisms

Several underlying mechanisms have been suggested to explain the observed association between higher meat intake and increased risk of cancer and coronary heart disease (CHD) in the existing literature. For cancer, the exact mechanisms are not known but might be attributable to the formation of the mutagenic compounds polycyclic aromatic hydrocarbons (PAHs), heterocyclic amines (HCAs), and N-nitroso compounds (NOCs). PAHs and HCAs are formed during cooking of meat at high temperatures (31), and NOCs may be found in foods processed by smoking or in processed meat containing nitrite. Both PAHs, HCAs, and NOCs have been mechanistically linked to colorectal cancer (32).

It has been suggested that the content of fats in meat may be an explanation for the observed risk of CHD, but the total evidence suggests that other components of meat that are most relevant for cardiometabolic effects (33). The high content of salt in processed meat is suggested to account for a substantial proportion of the observed risk of CHD, due to the effects of sodium on blood pressure, and the observed relationship between blood pressure and clinical events of CHD (33). It has also been suggested that the mechanisms linking red and processed meat to the observed risk of CHD are the same as the suggested mechanisms linking red and processed meat to the observed risk of type 2 diabetes mellitus (34). However, the exact mechanisms for CHD are not clear and should be further investigated.

### Strengths and Limitations

The large sample size and long follow-up are some of the main strengths of the current study. The study population consisted of patients in Western-Norway with established coronary artery disease, and it has previously been reported that the participants in WENBIT are comparable to samples of patients with verified coronary artery disease in Europe (14). However, generalizability toward the general population is limited. To identify confounders, we used Directed acyclic graphs, which is a widely used tool for causal inference. Nevertheless, the current study was an observational study, and, therefore, residual confounding cannot be excluded. Also, dietary intake is associated with several social and behavioral confounders, such as health-consciousness, physical activity, and socioeconomic status that we did not measure. Furthermore, all patients received standard dietary advice for patients with cardiovascular disease when they were included in the study. Most patients conducted the FFQ after they had received these dietary advices, which may have affected how they responded to the questionnaire. As a dietary assessment was conducted only on the first visit, we do not know whether the patients changed their diet during follow-up. Dietary data derived from FFQs are also known to be affected by substantial measurement error, and measurement error in meat intake may lead to regression dilution bias and loss of statistical power to detect a relationship (35). However, we adjusted for self-reported energy intake using the nutrient density method, and by including self-reported total energy intake as a covariate in the multivariate models, which helps to attenuate the measurement error and improves precision when estimating diet-disease relationships (21, 36). The present FFQ did not allow for distinguishment between different types of meat. However, a comparison with the national dietary survey from 1997, “Norkost 2” (37) shows that the meat intake in this study population was at the same level as the general population in Norway. Food balance sheets from Norway (38) show that red meat dominated the meat intake in 1999, while white meat was only a small proportion.

In conclusion, in patients with stable angina pectoris, the association of a higher total meat intake with mortality and morbidity were in general inconclusive but indicated an increased risk of AMI and gastrointestinal cancer, particularly at higher meat intakes. The associations with all-cause mortality and AMI differed across ages, with increased risk observed at younger ages, but an attenuation, and even reversal of the risk association with increasing age. The findings from this study support the current dietary guidelines emphasizing a restricted meat intake in patients with CVD but highlight the need for more research on the association between meat intake and health outcomes in elderly populations. Future studies should investigate different types of meat separately in other CVD-cohorts, in different age-groups, as well as in the general population.

## Data Availability Statement

The raw data supporting the conclusions of this article will be made available by the authors, without undue reservation.

## Ethics Statement

The studies involving human participants were reviewed and approved by the Regional Committee for Health Research Ethics and the Norwegian Data Inspectorate. The patients/participants provided their written informed consent to participate in this study.

## Author Contributions

ÅM and VL analyzed data. ÅM had primary responsibility for final content and wrote the paper. AV, TO, TH, CB, ON, JD, and VL reviewed and edited the manuscript. All authors have read and approved the final version of the manuscript.

## Conflict of Interest

The authors declare that the research was conducted in the absence of any commercial or financial relationships that could be construed as a potential conflict of interest.

## Abbreviations

AMI: Acute myocardial infarction
CHD: Coronary heart disease
CVD: Cardiovascular disease
E%: Energy %
FFQ: Food frequency questionnaire
GI: Gastrointestinal
HCA: Heterocyclic amines
ICD: International classifications of diseases
NOC: N-nitroso compound
PAH: Polycyclic aromatic hydrocarbons
WENBIT: Western Norway B-vitamin Intervention Trial.
