# Supplementary material for: The Association of Meat Intake With All-Cause Mortality and Acute Myocardial Infarction Is Age-Dependent in Patients With Stable Angina Pectoris
Source: Front Nutr. 2021 Mar 4;8:642612. doi: 10.3389/fnut.2021.642612 (PMC7969515; doi:10.3389/fnut.2021.642612)
Supplement: Supplementary file 1 [file Data_Sheet_1.PDF]

Supplementary material

Supplemental Table 1 – Complete patient characteristics (n = 1929)

Variable	Association with meat intake[1]	Mean ± SD / n (%)	gMean ± gSD	Range	Percentiles					Gini's mean difference
					10	25	50	75	90	
Age, years	r = -0.20 (-0.24, -0.16)	61.8 ± 9.7	61.0 ± 1.18	28.0 – 85.0	49.0	55.0	62.0	69.0	75.0	11.1
BMI, kg/m^2	r = 0.14 (0.10, 0.18)	26.4 ± 3.7	26.1 ± 1.15	16 – 50	22.0	24.0	26.0	28.0	31.0	4.04
Male sex	d = 3.6 (1.1, 6.1)	1539 (79.8%)	-	-	-	-	-	-	-	-
Statin use	d = 1.3 (-1.9, 4.6)	1721 (89.2%)	-	-	-	-	-	-	-	-
Previous AMI	d = -0.3 (-2.4, 1.7)	836 (43.3%)	-	-	-	-	-	-	-	-
Waist circumference, cm	r = 0.12 (0.08, 0.17)	96.4 ± 11.1	95.7 ± 1.12	52.0 – 175	83.0	90.0	96.0	103	110	12.2
HbA1c, %	r = 0.04 (-0.00, 0.09)	5.97 ± 1.36	5.82 ± 1.24	1.40 – 16.1	4.49	5.12	5.81	6.58	7.51	1.43
**Blood lipids, mmol/L**										
Total-C	r = 0.06 (0.02, 0.11)	5.02 ± 1.19	4.90 ± 1.24	2.40 - 22.6	3.80	4.20	4.90	5.60	6.50	1.24
LDL-C	r = 0.03 (-0.01, 0.08)	3.04 ±1.01	2.88 ± 1.39	0.43 - 10.0	1.93	2.30	2.85	3.60	4.40	1.10
HDL-C	r = -0.01 (-0.06, 0.03)	1.26 ± 0.34	1.22 ± 1.40	0.00 - 3.00	0.9	1.00	1.20	1.42	1.70	0.37

Triglycerides	r = 0.09 (0.05, 0.14)	1.79 ± 1.15	1.57 ± 1.64	0.38 - 29.2	0.84	1.10	1.54	2.20	2.99	1.02
**Inflammation marker and renal function**										
CRP, mg/L	r = 0.05 (0.00, 0.09)	3.26 ± 6.35	1.69 ± 2.97	0.00 - 135	0.45	0.81	1.65	3.25	6.69	3.89
eGFR, mL/min	r = 0.11 (0.07, 0.16)	89.7 ± 15.5	88.1 ± 1.22	21.0 - 136	69.0	81.0	92.0	100	107	16.9
**Coronary risk factors**										
Hypertension	d = -0.4 (-2.5, 1.6)	911 (47.2%)	-	-	-	-	-	-	-	-
Diabetes[2]	d = 3.5 (1.3, 5.7)	590 (30.6%)	-	-	-	-	-	-	-	-
Current smoker[3]	d = 6.4 (4.2, 8.7)	560 (29.0%)	-	-	-	-	-	-	-	-
**Extent of CAD**										
Zero-vessel disease	d = 3.1 (-0.1, 6.3)	218 (11.3%)	-	-	-	-	-	-	-	-
One-vessel disease	d = 1.7 (-0.6, 3.9)	543 (28.1%)	-	-	-	-	-	-	-	-
Two-vessel disease	d = 1.3 (-1.0, 3.6)	533 (27.6%)	-	-	-	-	-	-	-	-
Three-vessel disease	d = -4.1 (-6.3, -1.9)	635 (32.9%)	-	-	-	-	-	-	-	-

[1] Given as d = mean difference (95% CI), or r = Pearson's correlation coefficient (95% CI), calculated on log transformed data

[2] Preexisting diagnose of diabetes, or with fasting blood glucose > 7 mmol/L, non-fasting blood-glucose > 11.1 mmol/L, or HbA1c > 6.5%

[3] Self-reported current smokers, or having quit within the last four weeks, or with plasma cotinine levels ≥ 85 nmol/L. Abbreviations: BMI, Body mass index; CRP, C-reactive protein; GFR, Glomerular filtration rate; gMean, Geometric mean; gSD, Geometric standard deviation; HDL-C, High-density lipoprotein - cholesterol; LDL, Low-density lipoprotein-cholesterol; TG, Triglycerides; Total-C, Total-cholesterol

Supplemental Table 2 – Complete description of the daily dietary intake of the patients (n = 1929)

Variable	Association with meat intake[1]	Mean ± SD	gMean ± gSD	Null consumers (%)	Range	Percentiles					Gini's mean difference
						10	25	50	75	90	
**Macronutrients (E%)**											
Fat	r = 0.29 (0.25, 0.33)	32.0 ± 5.5	31.5 ± 1.20	-	14.0 – 53.4	24.9	28.4	31.8	35.7	38.9	6.19
SFA	r = 0.23 (0.19, 0.27)	11.8 ± 2.6	11.5 ± 1.25	-	4.20 – 27.2	8.56	10.0	11.6	13.3	15.0	2.89
MUFA	r = 0.38 (0.35, 0.42)	10.3 ± 2.0	10.1 ± 1.22	-	4.23 – 18.3	7.86	8.97	10.3	11.6	12.8	2.22
PUFA	r = 0.12 (0.07, 0.16)	7.22 ± 1.96	6.97 ± 1.31	-	2.93 – 16.5	5.01	5.76	6.90	8.40	9.82	2.17
Carbohydrate	r = -0.36 (-0.40, -0.32)	49.1 ± 6.2	48.7 ± 1.14	-	23.0 – 75.9	41.3	45.1	49.3	53.4	56.7	6.90
Protein	r = 0.24 (0.20, 0.29)	16.7 ± 2.5	16.5 ± 1.16	-	8.92 – 29.2	13.7	15.0	16.5	18.2	19.9	2.78
Alcohol	r = 0.12 (0.08, 0.17)	1.70 ± 2.08	0.11 ± 71.8	493 (25.6%)	0 – 9.87	0.00	0.00	0.96	2.70	4.72	2.12
**Food groups, g/1000 kcal**											
Meat	-	54.9 ± 22.9	49.1 ± 1.80	1 (0.1%)	0 – 153	26.7	39.0	53.3	68.9	85.1	25.6
Egg	r = 0.07 (0.03, 0.12)	8.34 ± 6.12	5.00 ± 6.28	42 (2.2%)	0 – 54.4	1.96	4.03	7.15	11.0	15.9	6.42
Dairy	r = -0.09 (-0.13, -0.05)	158 ± 108	126 ± 2.44	1 (0.1%)	0 – 686	31.1	73.8	143	221	302	119

Milk	r = -0.05 (-0.10, -0.01)	133 ±107	75.1 ± 4.88	10 (0.5%)	0 – 681	11.3	41.1	118	194	271	117
Cheese	r = -0.00 (-0.05, 0.04)	13.3 ± 11.8	4.22 ± 23.6	141 (7.3%)	0 – 89.3	1.02	5.07	10.3	18.2	28.9	12.3
Bread	r = -0.09 (-0.13, -0.05)	92.6 ± 31.9	81.4 ± 2.87	10 (0.5%)	0 – 230	54.2	71.5	90.4	112	135	35.6
Grains	r = 0.02 (-0.02, 0.07)	15.8 ± 13.1	10.4 ± 3.69	11 (0.6%)	0 – 141	3.25	6.73	12.8	21.3	31.0	13.1
Fish	r = -0.03 (-0.08, 0.01)	53.7 ± 28.5	44.5 ± 2.46	5 (0.3%)	0 - 246	21.9	33.6	48.8	68.7	91.6	30.9
Vegetables	r = 0.08 (0.04, 0.13)	106 ± 75	84.3 ± 2.28	2 (0.1%)	0 - 942	37.4	57.5	88.7	134	189	73.1
Fruits and berries	r = -0.11 (-0.15, -0.06)	126 ± 86	97.8 ± 2.55	3 (0.2%)	0 - 913	40.9	67.9	108	163	230	88.4
**Other**											
Energy, kcal	r = 0.06 (0.02, 0.11)	2092 ± 631	1996 ± 1.37	-	721 - 4153	1320	1653	2033	2478	2953	809
Fiber, g/1000 kcal	r = -0.11 (-0.15, -0.06)	25.2 ± 8.6	23.7 ± 1.42	-	6.50 – 84.5	15.1	19.1	24.4	30.0	36.3	9.43
Cholesterol, mg/1000 kcal	r = 0.28 (0.24, 0.32)	142 ± 39	137 ± 1.31	-	43.7 - 338	98.2	116	138	162	189	42.1

[1] Given as r = Pearson's correlation coefficient (95% CI), calculated on log-transformed data

Abbreviations: gMean, Geometric mean; gSD, geometric standard deviation; MUFA, Monounsaturated fatty acid; PUFA, Polyunsaturated fatty acid; SFA, Saturated fatty acid

Supplemental Table 3 – Sex-specific associations between energy-adjusted total meat intake and clinical endpoints– results from the Cox proportional hazard regression model[1]

	All-cause mortality		AMI		Cancer		GI-cancer	
**Females**								
**Number of events** (%)	109 (27.9%)		64 (16.9%)		37 (9.5%)		13 (3.3%)	
**Models[2]**	**HR (95% CI)**	***P-value***	**HR (95% CI)**	***P-value***	**HR (95% CI)**	***P-value***	**HR (95% CI)**	***P-value***
Model 1	0.65 (0.39, 1.07)	*0.088*	1.19 (0.66, 2.16)	*0.563*	0.68 (0.29, 1.59)	*0.376*	0.75 (0.19, 3.02)	*0.690*
Model 2	0.66 (0.39, 1.10)	*0.109*	1.24 (0.67, 2.29)	*0.492*	0.70 (0.29, 1.68)	*0.426*	0.59 (0.14, 2.50)	*0.478*
Model 3	0.62 (0.37, 1.04)	*0.068*	1.14 (0.62, 2.09)	*0.673*	0.74 (0.32, 1.70)	*0.473*	0.78 (0.20, 3.00)	*0.722*
Model 4	0.67 (0.39, 1.14)	*0.136*	1.26 (0.67, 2.37)	*0.477*	0.75 (0.31, 1.80)	*0.520*	0.54 (0.13, 2.24)	*0.393*
**Males**								
	**All-cause mortality**		**AMI**		**Cancer**		**GI-cancer**	
**Number of events (%)**	465 (30.2%)		245 (15.9%)		176 (11.4%)		48 (3.1%)	
**Models[2]**	**HR (95% CI)**	***P-value***	**HR (95% CI)**	***P-value***	**HR (95% CI)**	***P-value***	**HR (95% CI)**	***P-value***
Model 1	0.97 (0.79, 1.20)	*0.748*	1.27 (0.96, 1.68)	*0.091*	1.12 (0.80, 1.57)	*0.505*	1.38 (0.74, 2.57)	*0.313*

Model 2	0.93 (0.75, 1.15)	*0.486*	1.20 (0.91, 1.59)	*0.198*	1.11 (0.79, 1.55)	*0.553*	1.34 (0.72, 2.52)	*0.358*
Model 3	0.95 (0.77, 1.18)	*0.656*	1.26 (0.95, 1.67)	*0.110*	1.12 (0.80, 1.56)	*0.509*	1.38 (0.74, 2.56)	*0.309*
Model 4	0.92 (0.74, 1.14)	*0.434*	1.19 (0.90, 1.59)	*0.231*	1.11 (0.79, 1.56)	*0.544*	1.37 (0.72, 2.59)	*0.333*

[1] HRs (95% CI) are given per daily 50 g/1000 kcal higher intake of meat. [2] Model 1: Adjusted for age, sex, smoking, and total energy intake. Model 2: Adjusted for age, sex, smoking, BMI, and total energy intake. Model 3: Adjusted for age (spline), sex, smoking, and total energy intake. Model 4: Adjusted for age (spline), sex, smoking, BMI (spline), and total energy intake. Abbreviations: AMI, Acute myocardial infarction; GI, Gastrointestinal

Supplemental Table 4 – Results from likelihood ratio tests comparing models with and without interaction term with age

		All-cause mortality		AMI		Cancer		GI-cancer	
	**Interaction**	**No**	**Yes**	**No**	**Yes**	**No**	**Yes**	**No**	**Yes**
**Model1[1]**	**Log likelihood**	-3958,12	-3955,9	-2170,87	-2166,9	-1486,73	-1486,46	-418,34	418,00
	**Likelihood ratio[3]**	4,44		7,94		0,54		0,68	
	**P-value[4]**	0,035		0,005		0,469		0,411	
**Model 2[2]**	**Log likelihood**	-3955,41	-3953,15	-2169,22	-2165,24	-1486,70	-1486,44	-417,49	-417,14
	**Likelihood ratio[3]**	4,52		7,96		0,52		0,70	
	**P-value[4]**	0,034		0,005		0,468		0,405	

[1]Adjusted for age, sex, and smoking. [2]Adjusted for age, sex, smoking, and BMI. [3]Calculated as 2×(Log likelihood [Yes] – Log likelihood [No]).

[4]From Chi-squared test. Abbreviations: AMI, Acute myocardial infarction; GI, gastrointestinal

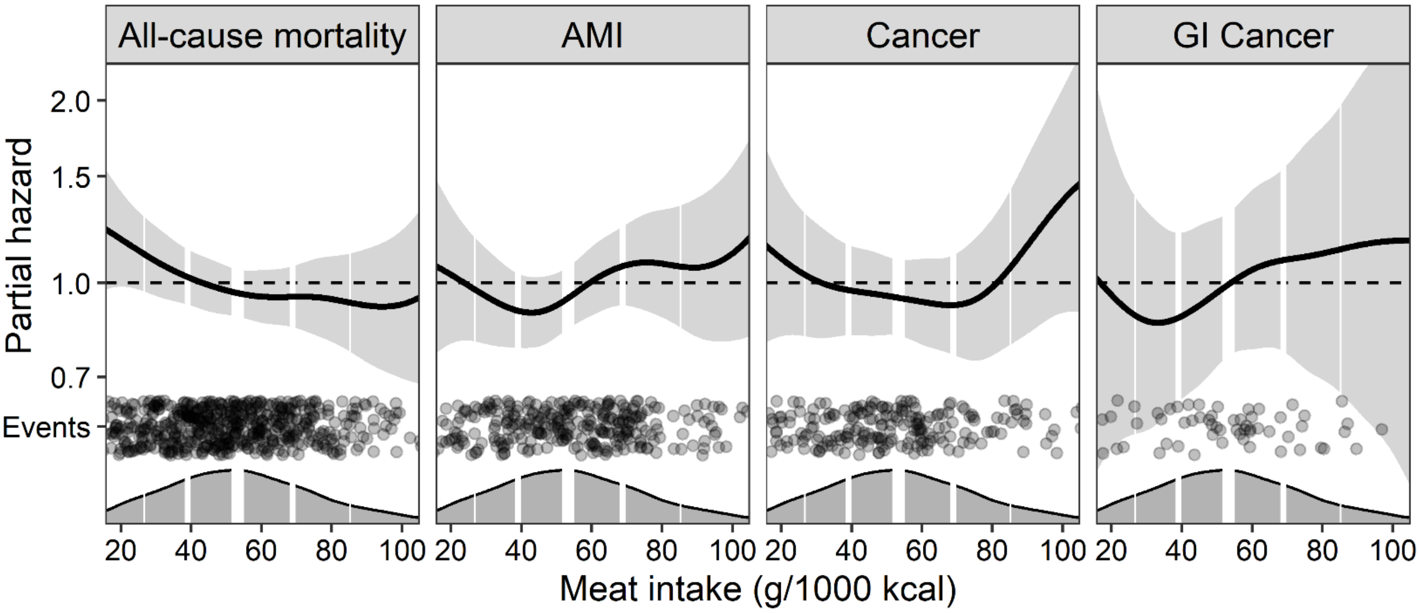

Supplemental Figure 1 – The continuous association between energy-adjusted total meat intake and the risk of clinical outcomes using generalized additive models. The models were adjusted for Model 2 covariates (self-reported energy intake, age, sex, BMI, and smoking)**.** Light grey areas around the central line represent 95% CIs of the hazard estimates. Dark grey areas at the x-axis are density plots of meat intake (g/1000 kcal), and the vertical white lines indicate the 10th, 25th, 50th (bold line),75th, and 90th percentiles. The black dots indicate events at the different intake levels of meat. The plots are cropped at the 2.5^{th} and 97.5^{th} percentiles of reported meat intake. Abbreviations: AMI, Acute myocardial infarction; GI, Gastrointestinal

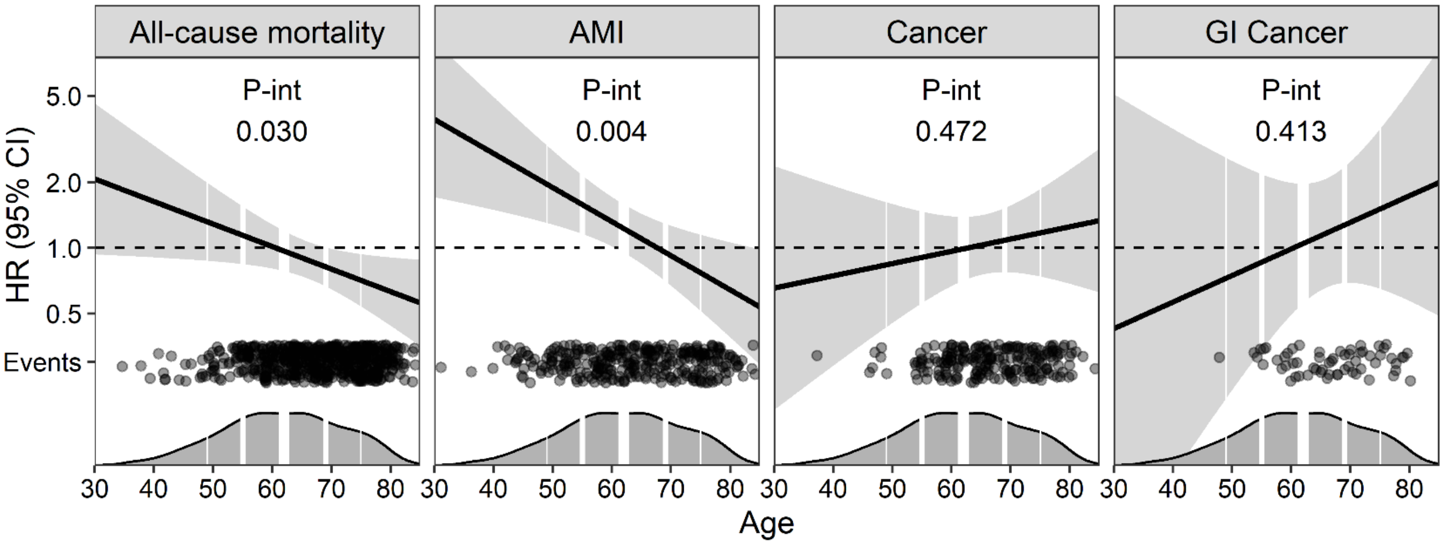

Supplemental Figure 2 – The association between energy-adjusted total meat intake and the risk of clinical outcomes across age. The curves are fitted by including age as an interaction term to Model 2 (adjusted for self-reported energy intake, age, sex, BMI, and smoking). The HRs are given per 50g/1000 kcal higher intake of meat, and light grey areas around the central line represent 95% CIs of the hazard ratios. Dark grey areas at the x-axis are density plots of age, and the vertical white lines indicate the 10th, 25th, 50th (bold line), 75th, and 90th percentiles. The black dots indicate events across age. Abbreviations: AMI, Acute myocardial infarction; GI, Gastrointestinal
