# Supplementary material for: The Association of Meat Intake With All-Cause Mortality and Acute Myocardial Infarction Is Age-Dependent in Patients With Stable Angina Pectoris
Source: Front Nutr. 2021 Mar 4;8:642612. doi: 10.3389/fnut.2021.642612 (PMC7969515; doi:10.3389/fnut.2021.642612)
Supplement: Supplementary file 2 [file Data_Sheet_2.PDF]

Analytic code

This file contains the code used to fit the survival models and to visualize the results. All analyses were performed in R version 4.0.3. The models were fitted using the *survival* package, and visualizations were made with *ggplot2* and *ggridges*.

Packages used

```
library(tidyverse)
library(broom)
library(survival)
library(ggridges)
```

Formatting data

- Making a nested data frame to apply the different models to the different outcomes

```
nested_data <- data %>%
  pivot_longer(names_to = "Outcome",
               values_to = "Status",
               cols = c(`All-cause mortality`, AMI, Cancer, `GI Cancer`)) %>%
  mutate(Time = case_when(Outcome == "All-cause mortality" ~ Time_death,
                          Outcome == "AMI" ~ Time_AMI,
                          Outcome == "Cancer" ~ Time_cancer,
                          Outcome == "GI Cancer" ~ Time_gi_cancer)) %>%
  mutate(across(Outcome, fct_inorder)) %>%
  group_by(Outcome) %>%
  nest()
```

Cox regression analyses

- Applying the different cox regression models on the nested tibble.
- Extracting HR and 95% CI per 50g/1000 kcal increment in meat intake

```
result_per50 <- nested_data %>%
  mutate(survobj = map(data, ~Surv(.x$Time, .x$Status == 1)),
         Model1 = map2(data, survobj, ~coxph(.y~Meat + Age + Sex + Smoking +
Kcal, data = .x)),
         Model2 = map2(data, survobj, ~coxph(.y~Meat + Age + Sex + Smoking +
Kcal + BMI, data = .x)),
         Model3 = map2(data, survobj, ~coxph(.y~Meat + pspline(Age) + Sex +
Smoking + Kcal, data = .x)),
         Model4 = map2(data, survobj, ~coxph(.y~Meat + pspline(Age) + Sex +
Smoking + Kcal + pspline(BMI), data = .x))) %>%
  pivot_longer(cols = starts_with("Model"),
               names_to = "Model",
               values_to = "Output") %>%
```

```
  mutate(Model = fct_inorder(Model)) %>%
  select(-data, -survobj) %>%
  mutate(HR = map_dbl(Output,  ~summary(.x, scale = 50)$conf.int[1,1] %>%
round(2)),
         Low = map_dbl(Output,  ~summary(.x, scale = 50)$conf.int[1,3] %>%
round(2)),
         High = map_dbl(Output,  ~summary(.x, scale = 50)$conf.int[1,4] %>%
round(2)))
```

- Format result table

```
nested_data %>% unnest(everything()) %>%
  summarise(result = paste0(sum(Status == 1), " (", round(100*sum(Status ==
1)/n(), 1) %>% sprintf(fmt = "%#.1f"), ")"), Model = "N events (%)") %>%
  remove_rownames() %>%
  bind_rows(result_per50 %>%
  mutate(result = paste0(HR, " (", Low, " - ", High, ")"),
         coef = map(Output, ~summary(.x)$coefficients),
         p = map_dbl(coef, ~.x[1,ncol(.x)] %>% round(3))) %>%
  select(Outcome, Model, result, p)) %>%
  pivot_wider(names_from = Outcome,
              values_from = c(result, p)) %>%
  select(Model,
         contains("All-cause"),
         contains("AMI"),
         contains("_Cancer"),
         contains("GI Cancer")) %>%
  rename_at(vars(starts_with("result")),
            ~str_replace(.x, "result_", ""))
```

Visualizing the continuous association

- Fitting a cox regression model with meat intake included as a spline using the *survival::pspline()* function, and plot the predicted values.
- The partial effects of meat was extracted using the *predict()* function
- Density plots to illustrate the distribution of meat intake were superimposed using the *ggridges::geom_density_ridges()*

```
nested_data %>%
  mutate(model = map(data, ~coxph(Surv(Time, Status == 1)~pspline(Meat) + Age
+ Sex + Smoking + Kcal, data = .x)),
         pred = map(model, ~predict(.x, type = "terms", term =
"pspline(Meat)", se.fit = T)),
         Meat = map(data, ~.x$Meat),
         HR = map(pred, ~exp(.x$fit)),
         Low = map(pred, ~exp(.x$fit - 1.96*.x$se.fit)),
         High = map(pred, ~exp(.x$fit + 1.96*.x$se.fit))) %>%
  select(-data, -model, -pred) %>%
  unnest(c(Meat, HR, Low, High)) %>%
  ggplot(aes(x = Meat)) +
  geom_smooth(aes(y = HR,
```

```
             ymin = Low,
             ymax = High),
             stat = "identity",
             color = "black") +
  geom_hline(yintercept = 1, lty = 2) +
  geom_density_ridges(aes(y = 0.4), scale = 5) +
  geom_vline(xintercept = quantile(data$Meat, c(0.10, 0.25, 0.5, 0.75,
0.90)),
             size = rep(c(0.3, 1, 2, 1, 0.3), 4),
             color = "white") +
  geom_smooth(aes(y = HR), stat = "identity", color = "black", se = F) +
  geom_jitter(data = eventdata %>% mutate(Event = 0.58), aes(y = Event),
              height = 0.06, alpha = 0.25) +
  facet_wrap(~Outcome, nrow = 1) +
  labs(x = "Meat intake (g/1000 kcal)",
       y = "Partial hazard") +
  coord_trans(y = "log",
              ylim = c(0.4, 2.3),
              xlim = c(quantile(data$Meat, 0.025), quantile(data$Meat,
0.975))) +
  scale_y_continuous(breaks = c(0.58, 0.7, 1, 1.5, 2),
                     labels = c("Events", "0.7", "1.0", "1.5", "2.0"),
                     expand = c(0,0)) +
  scale_x_continuous(breaks = c(20,40,60,80,100), expand = c(0,0)) +
  theme(axis.title.y = element_text(hjust = 0.7, vjust = -5))
```

Effect modification by age

- This code Creates a function to calculate all values necessary for plotting. Returns a tibble with HR and 95% CI for every age.

```
int_effects <- function(model, data, unit = 50, increment = 1){

# define variables, input data, and scale estimates/covariance matrix

exposure <- names(model$coefficients)[1]
moderator <- names(model$coefficients)[2]
interact <- paste0(exposure, ":", moderator)
covs <- vcov(model)
beta <- coef(model)

# calculate values
mlevels <- seq(min(data[moderator], na.rm = T), max(data[moderator], na.rm =
T), increment)
coefs <- beta[exposure] + beta[interact]*mlevels
se <- sqrt(covs[exposure, exposure] + mlevels^2*covs[interact, interact] +
2*mlevels*covs[exposure, interact])
HR <- exp(unit*coefs)
Low <- exp(unit*(coefs - 1.96*se))
High <- exp(unit*(coefs + 1.96*se))
```

```
# compile output

tibble(!!paste0(moderator) := mlevels,
       HR, Low, High)
}
```

- Create the models with the interaction term

```
intdata_1 <- nested_data %>%
  mutate(mod = map(data, ~coxph(Surv(Time, Status == 1)~Meat*Age + Sex +
Smoking + Kcal, data = .x)),
         int = map2(mod, data, ~int_effects(..1, ..2, unit = 50, increment =
0.1, plot = F)),
         pint = map_chr(mod, ~tidy(.x) %>%
                          tail(1) %>%
                          pull(p.value) %>%
                          round(3) %>%
                          sprintf("%.3f", .) %>%
                          gsub("0.000", "<0.001", .) %>%
                          paste0("P-int\n", .)),
         moderator = map(data, ~.x$Age),
         dist = map(data, ~.x$Age),
         event = map(data, ~case_when(as.numeric(as.character(.x$Status)) ==
0 ~ NA_real_,
                                      TRUE ~ 0.3)))
```

- Plot the data

```
intdata_1 %>%
  select(Outcome, int) %>%
  unnest(int) %>%
ggplot(aes(x = Age)) +
  geom_smooth(aes(y = HR,
                  ymin = Low,
                  ymax = High),
              stat = "identity",
              color = "black") +
  geom_hline(yintercept = 1, lty = 2) +
  facet_wrap(~Outcome, nrow = 1) +
  geom_density_ridges(data=intdata_1 %>% unnest(moderator),
                      aes(x = moderator, y = 0.1), scale = 2) +
  geom_vline(xintercept = quantile(data$Age, c(0.10, 0.25, 0.5, 0.75, 0.90)),
             size = rep(c(0.3, 1, 2, 1, 0.3), 4),
             color = "white") +
  geom_smooth(aes(y = HR),
              stat = "identity",
              color = "black", se = F) +
  geom_jitter(data = intdata_1 %>% unnest(c(moderator, event)),
              aes(x = moderator, y = event),
              height = 0.06, alpha = 0.4)+
  geom_text(data = intdata_1, aes(label = pint),
```

```
            x = 60, y = 6,
            hjust = 0.5, vjust = 1) +
  coord_trans(y = "log",
              ylim = c(0.1, 7.5),
              xlim = c(30,85)) +
  scale_x_continuous(expand = c(0,0)) +
  scale_y_continuous(breaks = c(0.3, 0.5, 1, 2, 5),
                     labels = c("Events", "0.5", "1.0", "2.0", "5.0"),
                     expand = c(0,0)) +
  labs(y = "HR (95% CI)") +
  theme(axis.title.y = element_text(hjust = 0.7, vjust = -5))
```

Compare cox regression models

- log likelihood extracted using *broom::glance()*
- likelihood ratio calculated as 2*(log likelihood difference)
- Chi^2 P-value obtained from the *anova()* function

```
nested_data %>%
  mutate(survobj = map(data, ~Surv(.x$Time, .x$Status == 1)),
         Model1 = map2(data, survobj, ~coxph(.y~Meat + Age + Sex + Smoking +
Kcal, data = .x)),
         Model1_interaction = map2(data, survobj, ~coxph(.y~Meat*Age + Sex +
Smoking + Kcal, data = .x)),
         Model2 = map2(data, survobj, ~coxph(.y~Meat + Age + Sex + Smoking +
Kcal + BMI, data = .x)),
         Model2_interaction = map2(data, survobj, ~coxph(.y~Meat*Age + Sex +
Smoking + Kcal + BMI, data = .x))) %>%
  pivot_longer(cols = c(Model1, Model2),
               names_to = "Modelname",
               values_to = "Basic") %>%
  pivot_longer(cols = c(Model1_interaction, Model2_interaction),
               names_to = "Model_int",
               values_to = "Interaction") %>%
  filter(str_detect(Model_int, Modelname)) %>%
  mutate(`Without interaction`= map_dbl(Basic, ~glance(.x) %>% pull(logLik)
%>% round(2)),
         `With interaction` = map_dbl(Interaction, ~glance(.x) %>%
pull(logLik) %>% round(2)),
         LR = 2*(`With interaction` - `Without interaction`) %>% round(2),
         P = map2_dbl(Basic, Interaction,
                      ~anova(.x, .y) %>%
                        tidy() %>%
                        tail(1) %>%
                        pull(p.value) %>%
                        round(3))) %>%
  select(-data, -survobj, -Model_int, -Basic, -Interaction)
```
